# Treatment of recurrent IgA nephropathy after kidney transplantation with targeted-release budesonide – a case report

**DOI:** 10.1007/s40620-025-02405-3

**Published:** 2025-09-12

**Authors:** Maximilian Packbiers, Annika Hahm, Theresa Riebeling, Jan Hinrich Bräsen, Roland Schmitt, Kevin Schulte

**Affiliations:** 1 Department of Nephrology and Hypertension, University Hospital Schleswig-Holstein, Christian-Albrechts-University, Arnold-Heller-Str. 3 Haus C, 24105 Kiel, Germany; 2 Hannover Med Sch, Inst Pathol, Nephropathol Unit, 30625 Hannover, Germany

**Keywords:** IgA nephropathy, Berger's disease, Kidney transplant, Budesonide

## Abstract

Immunoglobulin A nephropathy (IgAN) is one of the most common forms of primary glomerulonephritis which can lead to kidney failure requiring kidney replacement therapy via dialysis or transplantation. Unfortunately, IgAN can recur within the allograft. For treatment of primary IgAN, a targeted-release formulation of budesonide that acts specifically within the ileum can be used to prevent disease progression. The use of targeted-release budesonide in the setting of recurrent IgAN after transplantation has not yet been studied in detail. We here report a 28-year-old female with IgAN recurrence after transplantation, treated by targeted release budesonide for 9 months. Prior to treatment initiation in April 2023, estimated glomerular filtration rate (eGFR) drastically decreased, reaching  24 ml/min/1.73 m^2^ within 10 months. With treatment, the eGFR decrease slowed down considerably (− 6 ml/min/1.73 m^2^ within 12 months). The urine protein-to-creatinine-ratio (UPCR) likewise decreased from 4.55 g/g creatinine before therapy start to 1.30 g/g 12 months after therapy start. Despite episodes of poorly controlled hypertension and edema during treatment that were related to interruption of medications, blood pressure was stable at 122/77 mmHg after 9 months and 133/83 mmHg after 12 months. Compared to the beginning of the therapy, the patient lost 3 kg of body weight. There were no serious infections, nor was an increased susceptibility to infections observed. No other serious adverse events occurred. Although the patient experienced corticosteroid-related side effects, treatment was not interrupted. After therapy, the side effects subsided and the patient reports general wellbeing.

**Graphical Abstract:**

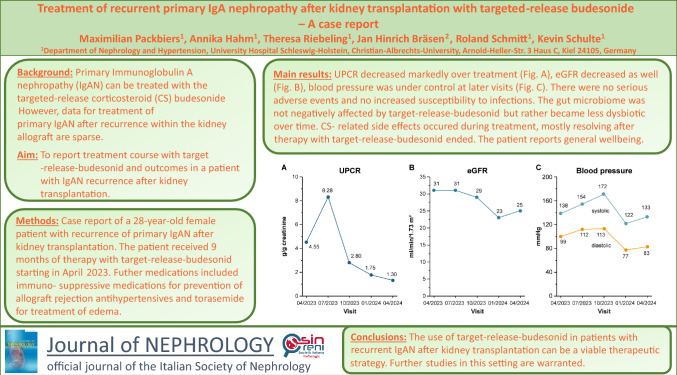

## Introduction

Immunoglobulin A nephropathy (IgAN) is one of the most common forms of primary glomerulonephritis, with an annual incidence of 0.76 per 100,000 people in Europe. [1] The central hallmark of IgAN is the (co-)deposition of IgA1 within kidney glomeruli. [1, 2] Usual symptoms are proteinuria, hypertension, decline of estimated glomerular filtration rate (eGFR) and kidney injury. [1, 3] Treatment of IgAN comprises vigorous blood pressure control and medications that treat proteinuria. [4]

Systemic immunosuppressive treatments, e.g., with corticosteroids have been used to reduce inflammation in IgAN. However, the European STOP-IgAN trial showed no benefit of oral corticosteroids vs. supportive care, while oral corticosteroids resulted in higher rates of adverse events. [5] The latter was also reported in the TESTING trial. [6] Therefore, a targeted-release formulation of budesonide was developed that acts directly in the Peyer’s patches–rich area within the ileum, where it downregulates the production of disease-associated forms of IgA1. [7] Targeted-release budesonide has shown promising efficacy regarding reduction of proteinuria and eGFR decline [8] and is approved for the treatment of patients with primary IgAN with a urine protein-to-creatinine ratio (UPCR) of ≥ 0.8 g/g creatinine or a 24 h proteinuria of 1.0 g, with a treatment duration of 9 months and a recommended dose of 16 mg once daily. [9]

IgAN is a progressive disease, so IgAN patients may develop kidney failure at some point. In a recent registry study, 28% reached a composite endpoint (eGFR decline > 40%, eGFR < 15 ml/min/1.73 m.^2^ and initiation of kidney replacement therapy) already after 6.5 years of follow-up. [10] When kidney failure is reached, kidney transplantation may become necessary. However, IgAN can recur and cause allograft failure, with a reported 10-year incidence of approximately 10% [11].

Although there are currently no established guideline recommendations for recurrent IgAN [11], targeted-release budesonide can be used as it is indicated for primary IgAN, regardless of the setting (initial occurrence/recurrence). To date, data for the use of targeted-release budesonide in the recurrent setting are sparse. We here report on the treatment course and results of treatment with targeted-release budesonide in a female patient with recurrent primary IgAN after kidney transplantation.

## Case report

**Fig. 1:**
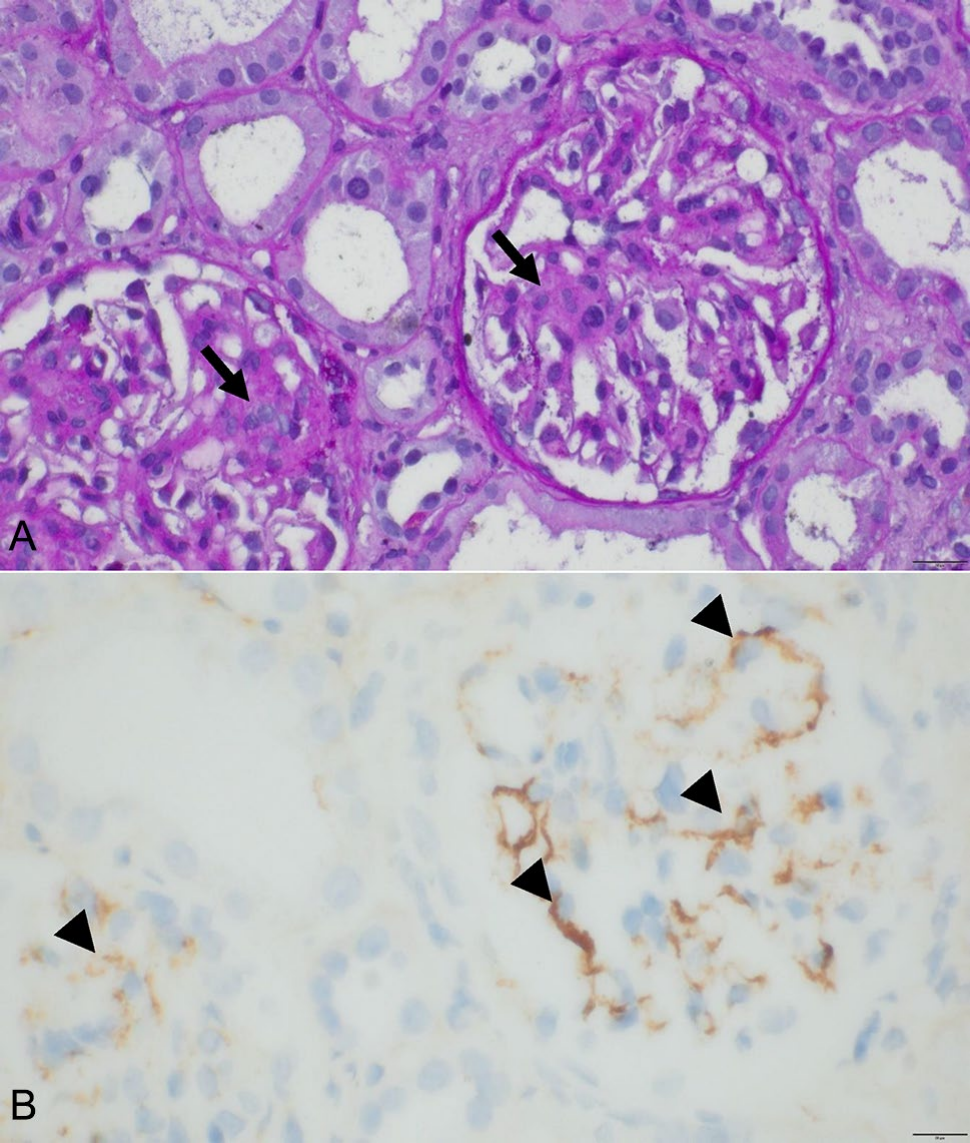
Histology of recurrent IgA-nephropathy in the transplanted kidney. Increase of mesangial cells can be seen (A, arrows) coinciding with positive IgA staining (B, arrowheads). Periodic Acid-Schiff (PAS) stain in (**A)**; immunohistochemistry for IgA visualized by DAB chromogen in (**B)**. Bars represent 20 micometers

The patient (female, 26 years old) had undergone kidney transplantation due to IgAN. Two years after the transplant, the eGFR dropped considerably from 50 ml/min/1.73 m^2^ (one year after transplant) to 26 ml/min/1.73 m^2^ (two years after transplant). Of note, eGFR was always calculated with the Chronic Kidney Disease Epidemiology Collaboration (CKD-EPI) formula. Due to the drop in eGFR, a biopsy was performed that confirmed IgAN recurrence (Banff score: i1 t1 v0 g0 ptc0 C4d0 ci1 ct1 cv0 cg0 ptcml0 ti2 i-IFTA3 t-IFTA1 pvl0 ah2 mm3; MEST-C score: M1 E0 S1 T0 C0; see Fig. 1). In the past, the patient had not well tolerated high-dose oral corticosteroids and cyclophosphamide did not provide benefit. Also taking into account the patient’s age, we decided against renewed administration of cyclophosphamide. The option of initiating therapy with targeted-release budesonide was discussed with the patient. Based on her UPCR of 4.55 g/g creatinine, the patient was eligible for therapy with targeted-release budesonide (Kinpeygo®, 16 mg once daily), so it was initiated and maintained for 9 months. Throughout the treatment period, the patient received immunosuppressive medications to prevent allograft rejection, including mycophenolate (1.440 mg daily), prednisolone (5 mg daily) and belatacept 400 mg (starting in May 2022, Q4W). For blood pressure control and treatment of chronic kidney disease, the patient received the following drugs (daily dose): diltiazem 180 mg, increased stepwise to 360 mg, ramipril 10 mg, spironolactone 25 mg and dapagliflozin 10 mg. Torasemide was administered in varying doses (5–20 mg) to correct edema. As the patient suffered from gastroesophageal reflux disease (GERD), pantoprazole in varying doses (20–80 mg) was administered.

There were follow-up visits every three months for 12 months. Several parameters were assessed at each visit and disease-related parameters included: adverse events (any), body weight, blood pressure, eGFR, UPCR, cortisol (serum and urine), IgA (serum) and gut microbiome status (dysbiosis index according to Casén et al., 2015 [12]).

**Fig. 2:**
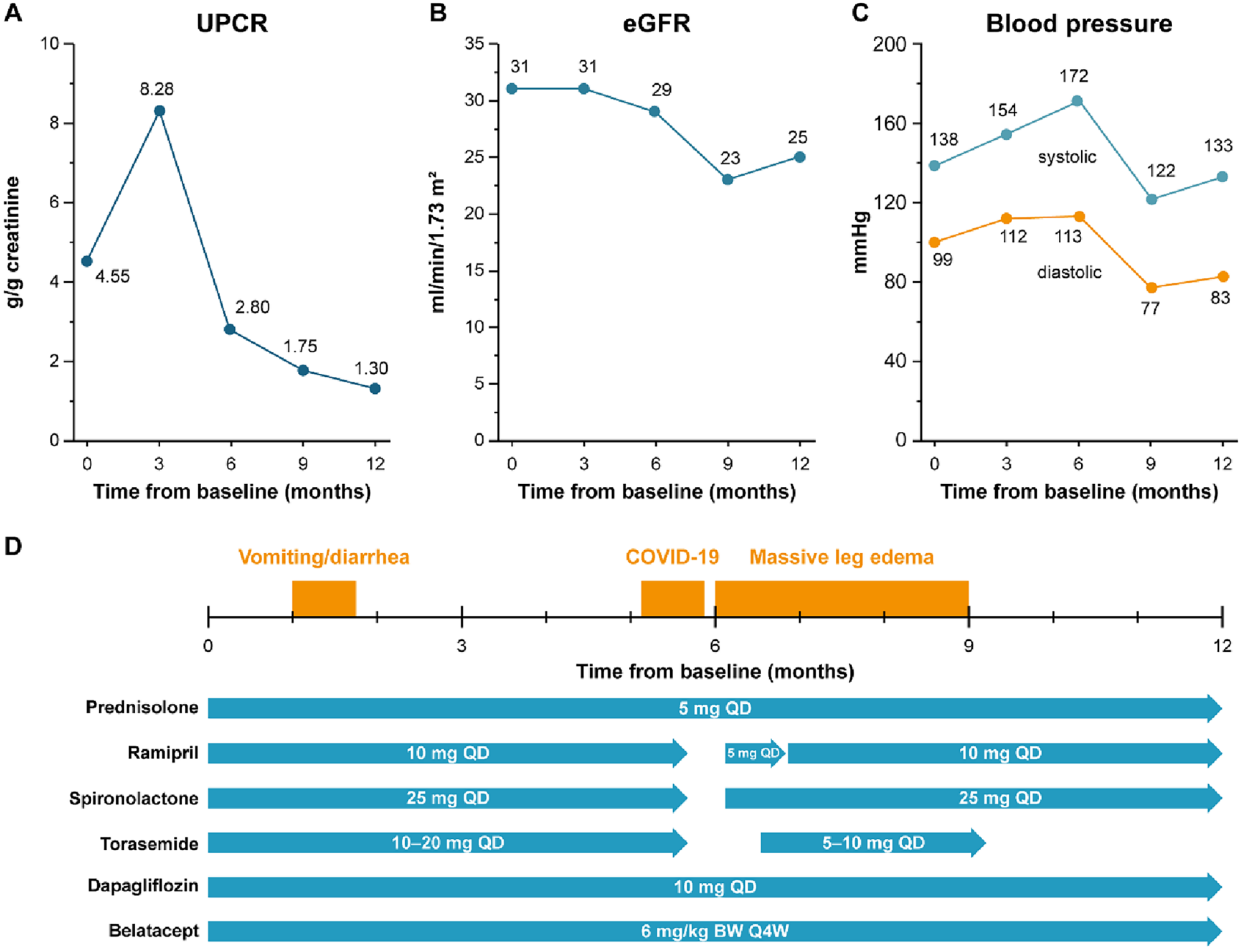
Overview of UPCR, eGFR, blood pressure, selected clinical events and concomitant medications during the treatment course with targeted-release budesonide. **A**: UPCR, **B**: eGFR, calculated by CKD-EPI formula, **C**: blood pressure, **D**: selected clinical events and concomitant medications. CKD-EPI: Chronic Kidney Disease Epidemiology Collaboration; eGFR: Estimated glomerular filtration rate; UPCR: Urine protein-to-creatinine ratio

The patient reported vomiting and diarrhea occurring one month after therapy start, which lasted three weeks. After three months, the patient had involuntarily gained weight (+ 2 kg) and experienced lower limb edema, therefore the torasemide dose was increased from 5 to 15 mg. The patient was advised to weigh herself regularly and to lower the torasemide dose as her weight decreased. Blood pressure was 154/112 mmHg, UPCR increased to 8.28 g/g creatinine, while eGFR remained stable at 31 ml/min/1.73 m^2^ (see Fig. 2A-C). An overview of selected clinical events and time course of selected concomitant medications is presented in Fig. 2D.

Five months after therapy start, the patient experienced a COVID-19 infection, lasting approximately 3 weeks, and manifesting with chills, cough, weakness, dyspnea and gastrointestinal problems. Because of the infection, torasemide, ramipril and spironolactone were interrupted and  were resumed after 4 weeks, while torasemide (5 mg) was resumed after 6 weeks. Six months after therapy start, the patient presented with reduced weight, but massive edema in the legs and strongly elevated blood pressure of 172/113 mmHg, possibly due to the interruption of medications one month prior. UPCR decreased markedly to 2.80 g/g creatinine and eGFR remained stable at 29 ml/min/1.73 m^2^ (see Fig. 2A-C). To control the high blood pressure, ramipril was dosed out to 10 mg and moxonidine was added (0.3 mg daily). Torasemide was increased slowly to 10 mg to treat the worsened leg edema. When asked about steroid-related side effects, the patient reported an increased incidence of hematomas, a swollen face, increasing facial hair, thinning head hair/hair loss, leg edema (especially after discontinuing torasemide) and extreme heartburn as well as an irregular menstrual cycle up to amenorrhea.

Nine months after therapy start, weight remained stable. Unfortunately, the leg edema did not improve. Blood pressure markedly improved to a daily mean of 122/72 mmHg through antihypertensive therapy with dosed out ramipril, diltiazem, moxonidine in maximal dosage and a loop diuretic. While UPCR decreased further to 1.75 g/g creatinine, eGFR decreased to 23 ml/min/1.73 m^2^ (see Fig. 2A-C). Regarding side effects, the patient reported general fatigue, persisting hematomas, swollen face, increased facial hair and thinning head hair/hair loss and amenorrhea, as well as petechia, and joint pain (in the knees, ankles and finger joints). The heartburn had improved considerably through a double dosage of pantoprazole. However, the patient still experienced bowel movement irregularities especially after physical exertion. Therapy with targeted-release budesonide ended after 9 months of continuous treatment.

The final visit to assess therapeutic outcome was carried out 12 months after therapy start, i.e., 3 months after the end of therapy with targeted-release budesonide. The patient lost weight (−1.4 kg compared to previous visit) and reported subjective wellbeing. Leg edema had reduced to low grade and blood pressure was in the normal to slightly elevated range with 133/83 mmHg (24 h measurement). Notably, UPCR decreased further to 1.30 g/g creatinine, eGFR increased slightly to 25 ml/min/1.73 m^2^ (see Fig. 2A-C).

Many of the steroid-related side effects that developed under therapy had improved markedly, including fatigue, petechia, bowel movement irregularities and joint pain, while heartburn, hair thinning and hematomas persisted.

When looking at further parameters (see Table 1), we observed that serum and urine cortisol decreased over the treatment period. Also, serum IgA decreased, and the gut microbiome became less dysbiotic over time.

**Table 1:** Change in selected parameters over the treatment period

Parameter	Baseline	3 months	6 months	9 months	12 months
Body weight (kg)	81.2	82.5	79.4	79.6	78.2
Cortisol (nmol/l)					
Serum	212	33	42	55	53
Urine	17.04	< 11.04	< 11.04	< 11.04	< 11.04
IgA (g/l), serum	1.84	1.56	1.74	1.33	1.32
Dysbiosis index [12]	–	4*	3	3	2

^*^Measurement after 4 months of treatment with targeted-release budesonide

*IgA* Immunoglobulin A

## Discussion

We here present the treatment results with targeted-release budesonide in a young female patient with IgAN recurrence after kidney transplantation. Data in this setting are sparse which highlights the practical relevance of our findings.

With targeted-release budesonide, UPCR decreased considerably from 4.55 g/g creatinine to 1.75 g/g after 9 months (−61.5%). This UPCR decrease also persisted 3 months after therapy (1.30 g/g creatinine, −71.4%) which is in line with results from the NefIgArd trial [8] and can be regarded as a success. In comparison, Lopez-Martinez et al. reported results for 5 transplanted IgAN patients treated with targeted-release budesonide and reported a UPCR decrease of 33.1% and 54.6% after 3 and 6 months, respectively. [13] However, contrary to our results, UPCR increased again after 12 months to −15.4% compared to baseline. [13]

Although eGFR decreased during the treatment period (–6 ml/min/1.73 m^2^, after 12 months), the decrease was considerably more drastic before therapy initiation (−24 ml/min/1.73 m.^2^ over 10 months), suggesting that the therapy slowed eGFR decline. Of note, eGFR 3 months and 6 months after therapy start might have been overestimated due to the high blood pressures, as an increased capillary pressure can result in increased net filtration and GFR [14].

The high blood pressure that occurred, especially after 6 months, was most likely a result of the interruption of blood pressure medications due to COVID-19. The medications were interrupted because of concerns that the patient might develop hypotension-related kidney damage caused by COVID-19 with diarrhea and the use of high-dose ramipril. After COVID-19 subsided and blood pressure medications were resumed, blood pressure returned to a normal range. In general, the patient did not exhibit an increased susceptibility to (serious) infections. This is an important finding, as increased susceptibility to infections is a major concern in transplanted patients under continuous corticosteroid therapy [15].

Regarding side effects, it was known that the patient poorly tolerated systemic corticosteroids. Considering this, the fact that therapy with targeted-release budesonide was not interrupted can be considered a success. In addition to targeted-release budesonide, the patient received 5 mg oral prednisolone to prevent allograft rejection. It has been demonstrated that 16 mg targeted-release budesonide leads to a systemic corticosteroid dose equivalent to 8 mg prednisolone [16]. This markedly higher systemic corticosteroid dose might have caused the reported corticosteroid-related side effects. It can be argued that oral prednisolone could have been halted during therapy with targeted-release budesonide to ameliorate side effects. In this regard, Gandolfini et al. analyzed 10 patients receiving targeted-release budesonide after kidney transplantation and found no benefit from therapy, concluding that there was no additional effect as patients were already on corticosteroids to prevent allograft rejection. [17] However, in this single-center study, a different formulation of targeted-release budesonide (that releases budesonide throughout the whole colon) was used, also the dosage and duration of use was lower [18].

In conclusion, the case presented here demonstrates that therapy with targeted-release budesonide can markedly reduce UPCR in recurrent IgAN after kidney transplantation. eGFR decreased, although the interpretation is difficult due to episodes of high blood pressure. While the additional corticosteroid therapy (in addition to oral prednisolone) led to an increase in corticosteroid-related side effects, it did not cause a higher susceptibility to infections, and the therapy was in general tolerable, without the occurrence of serious side effects. Further studies are warranted to analyze the efficacy and safety of targeted-release budesonide in patients with recurrent IgAN after kidney transplantation.

## Data Availability

Data will be made available upon reasonable request to the corresponding author.
